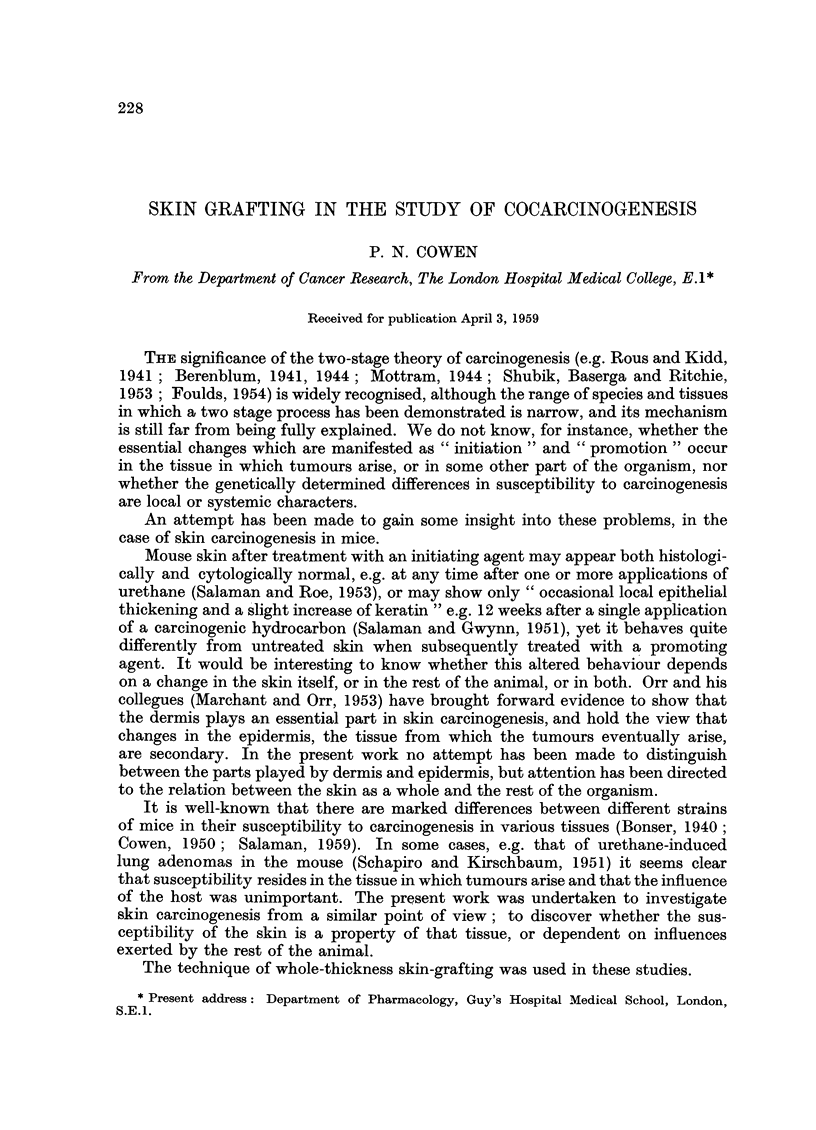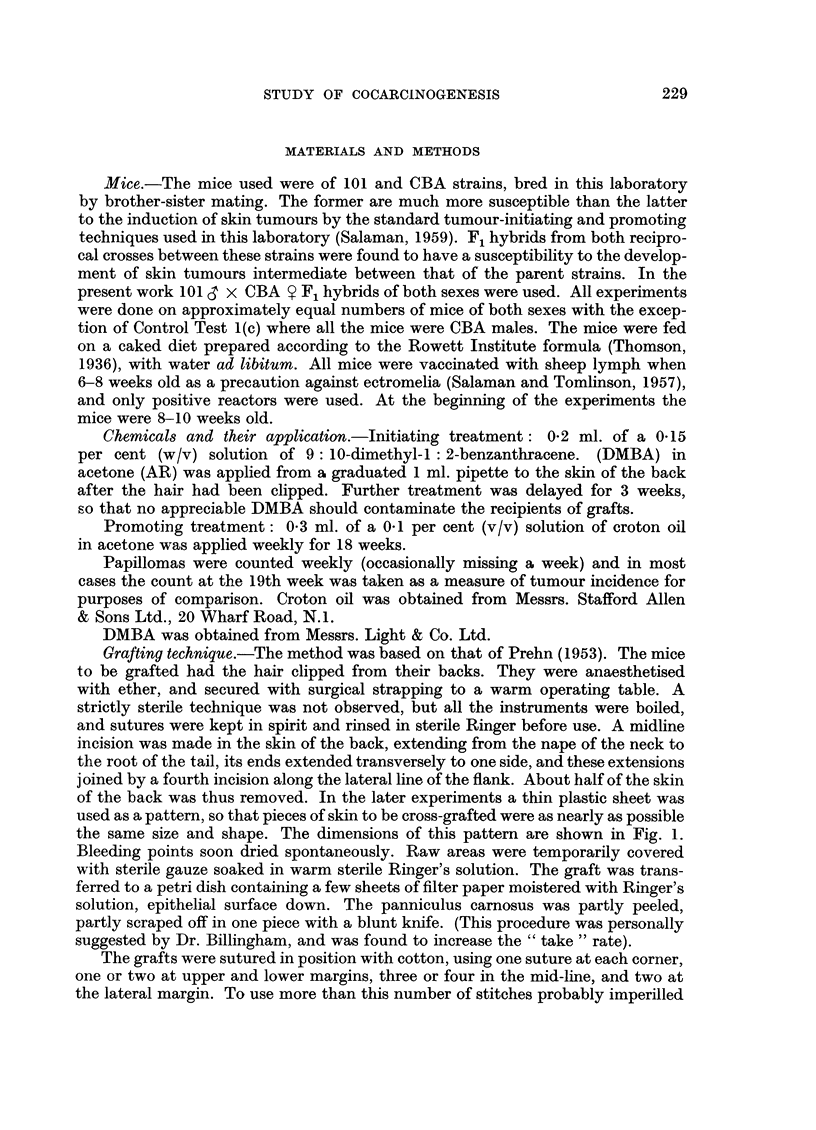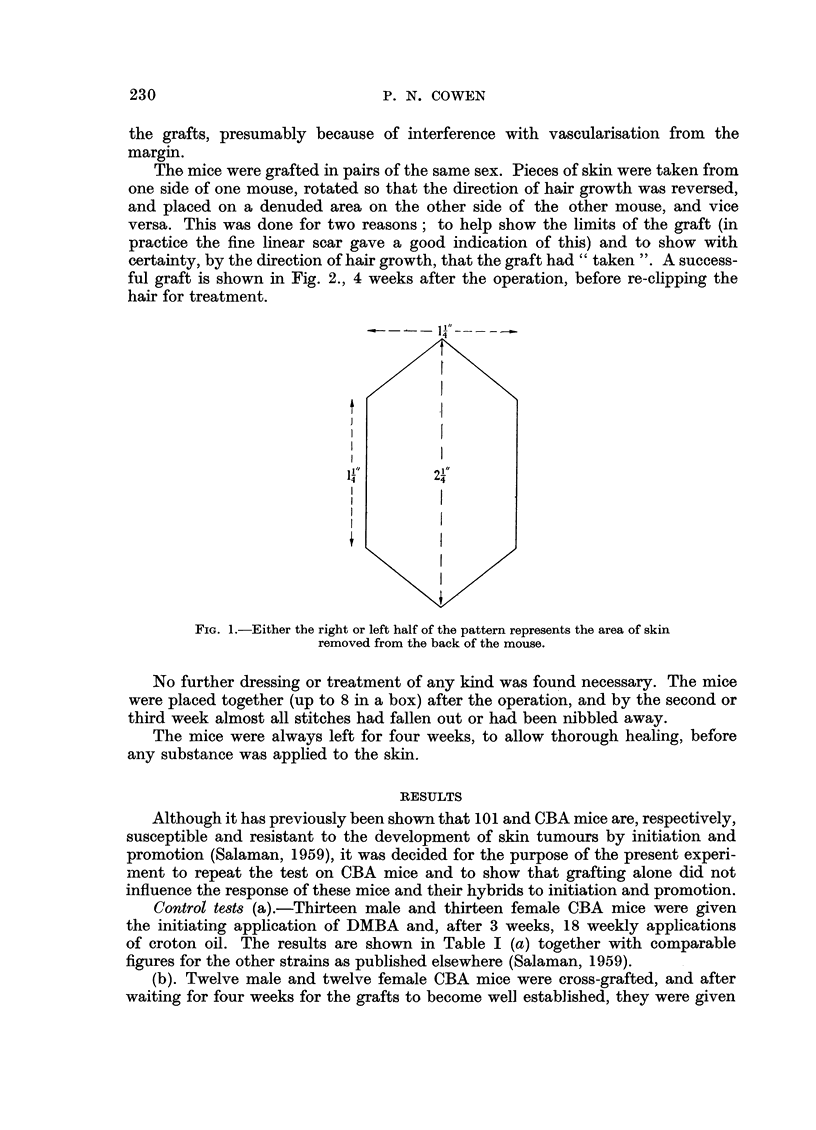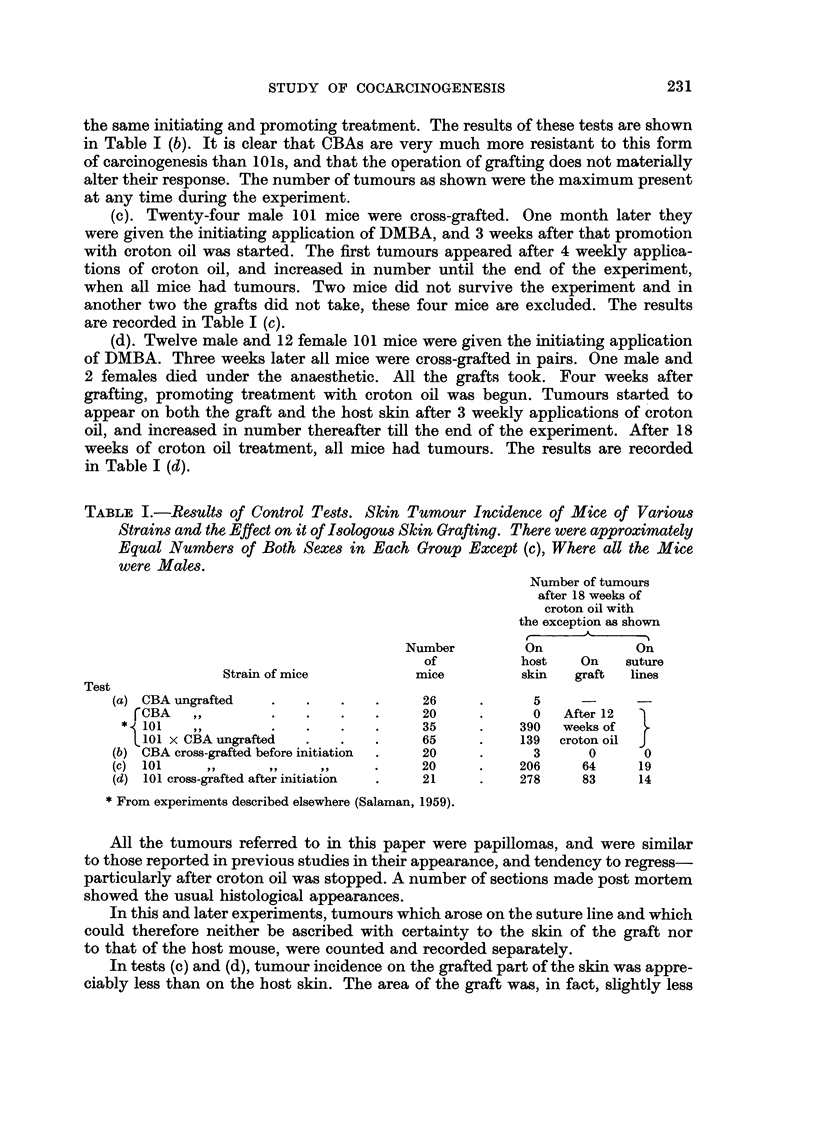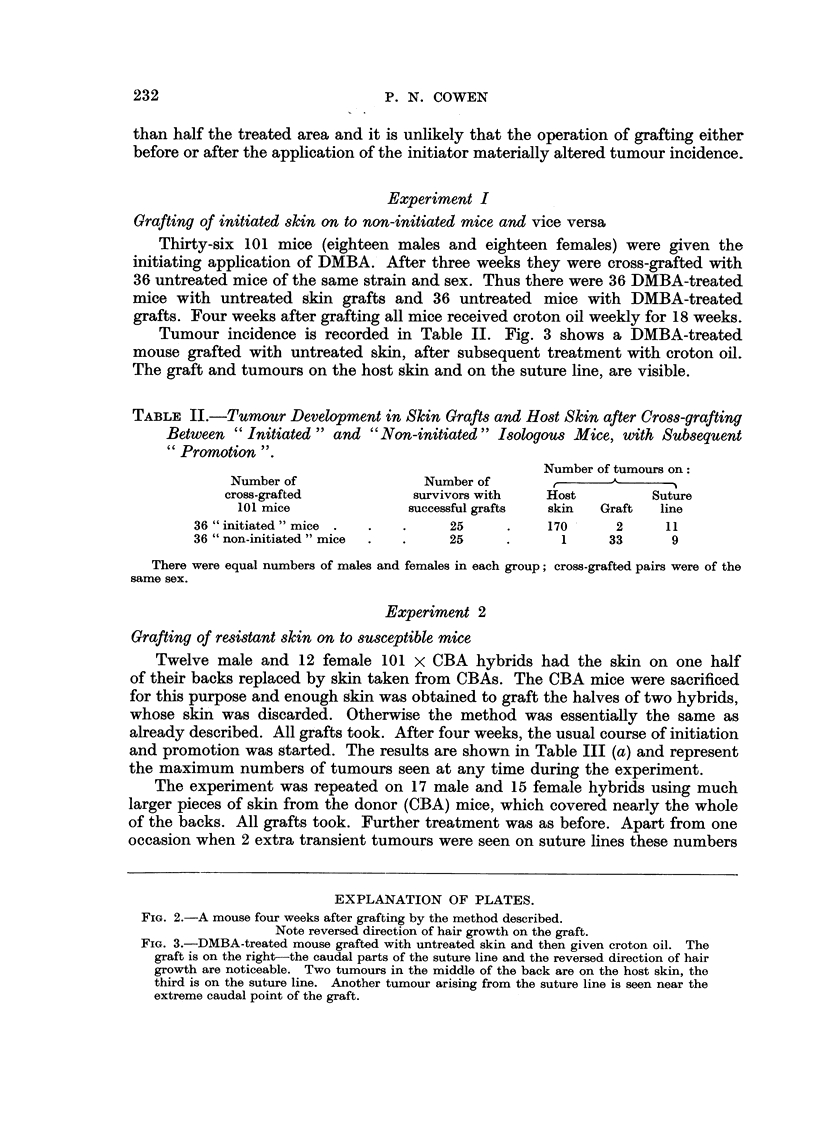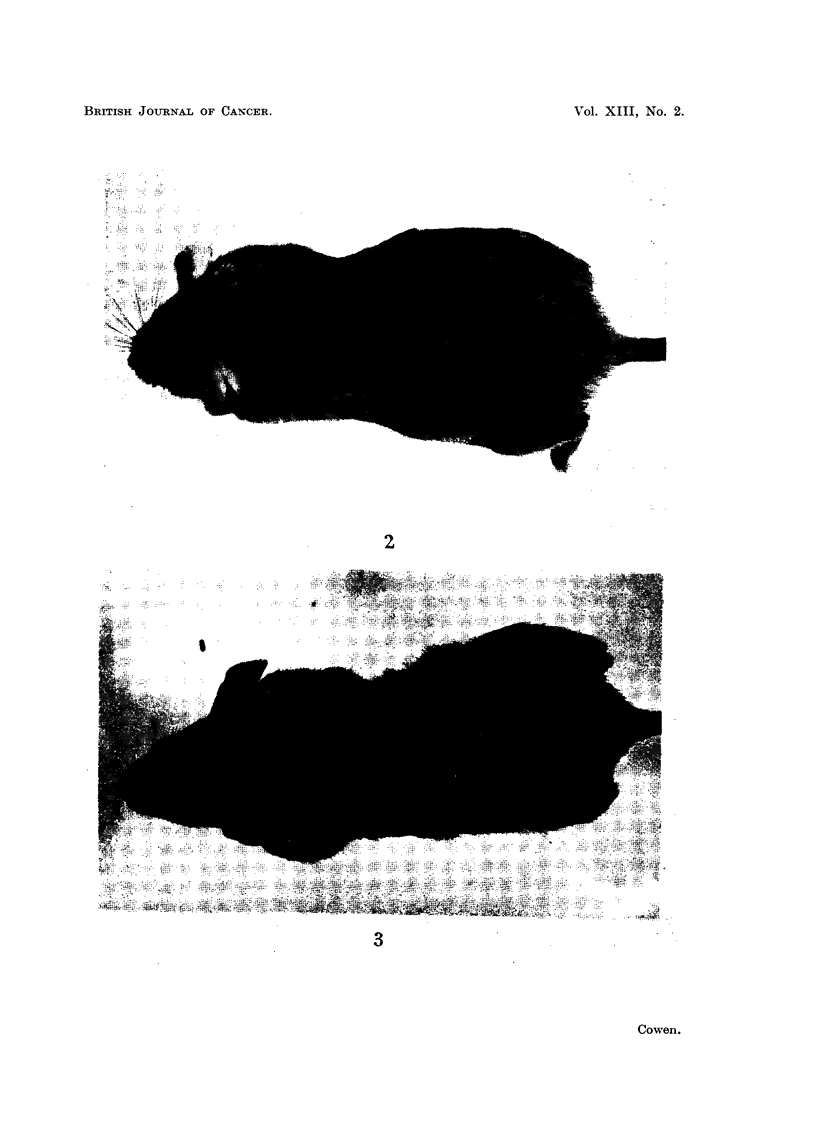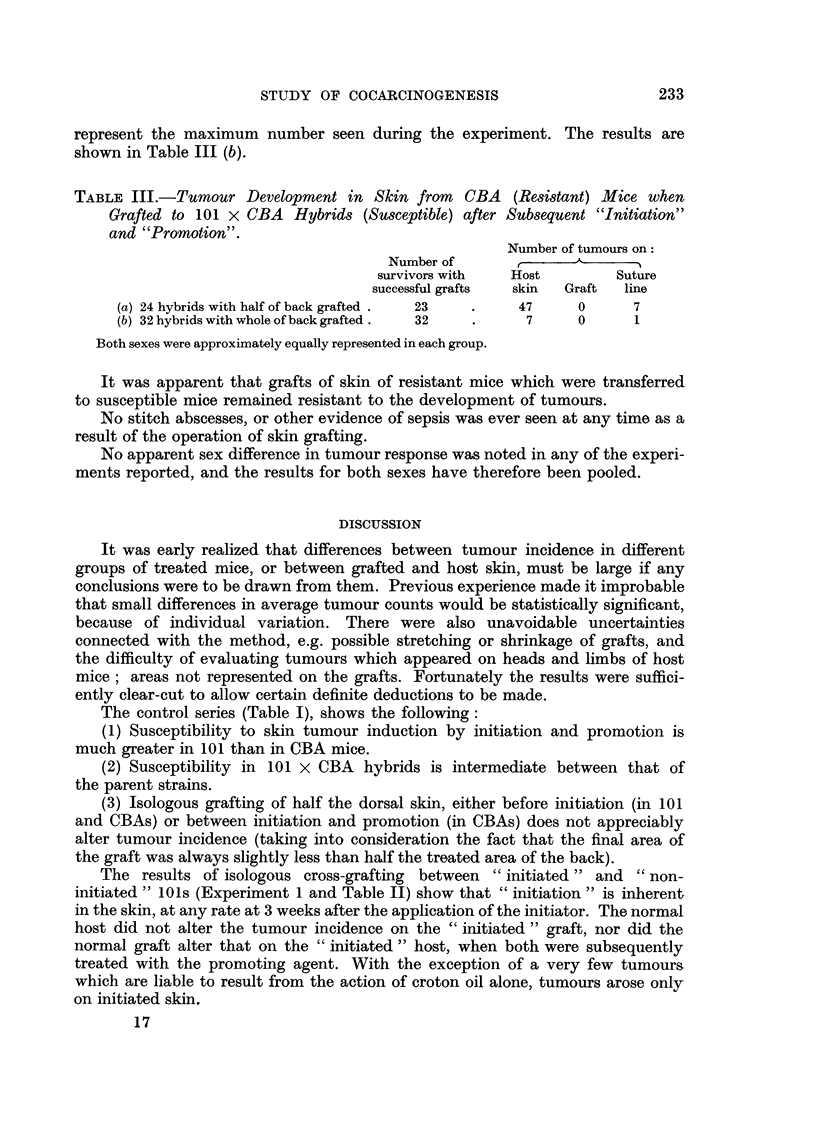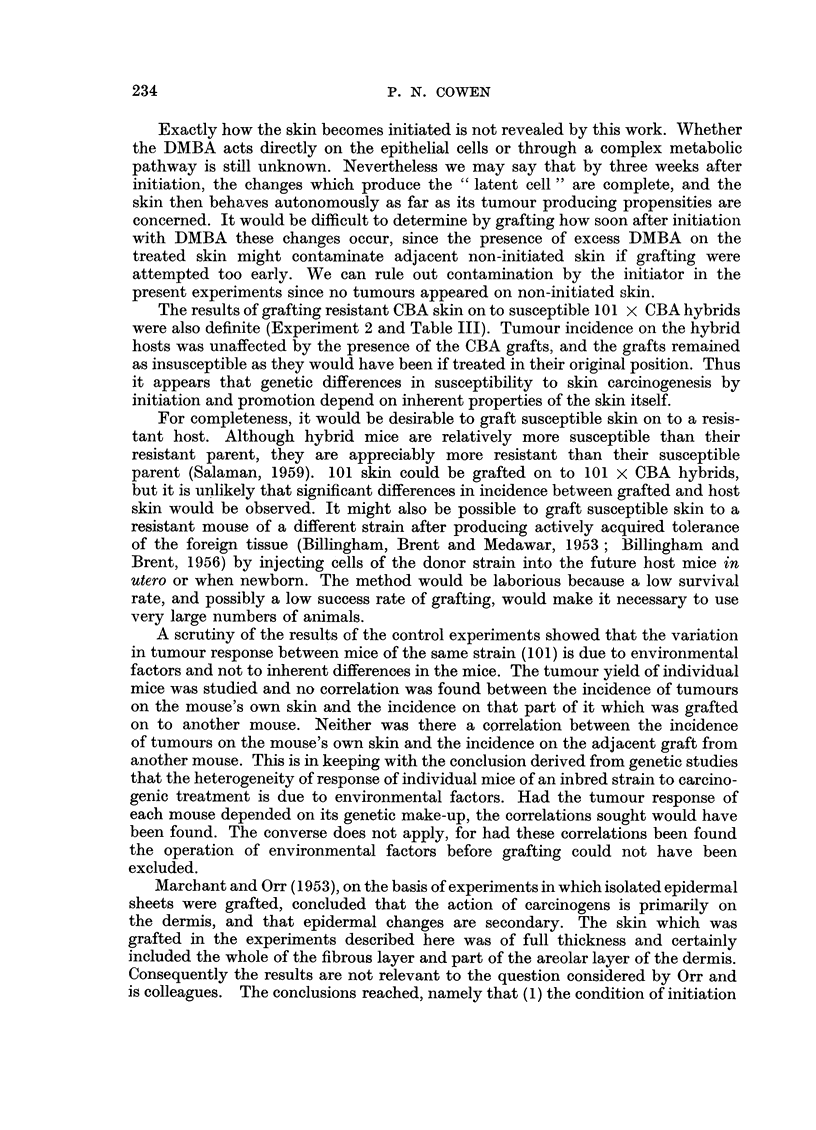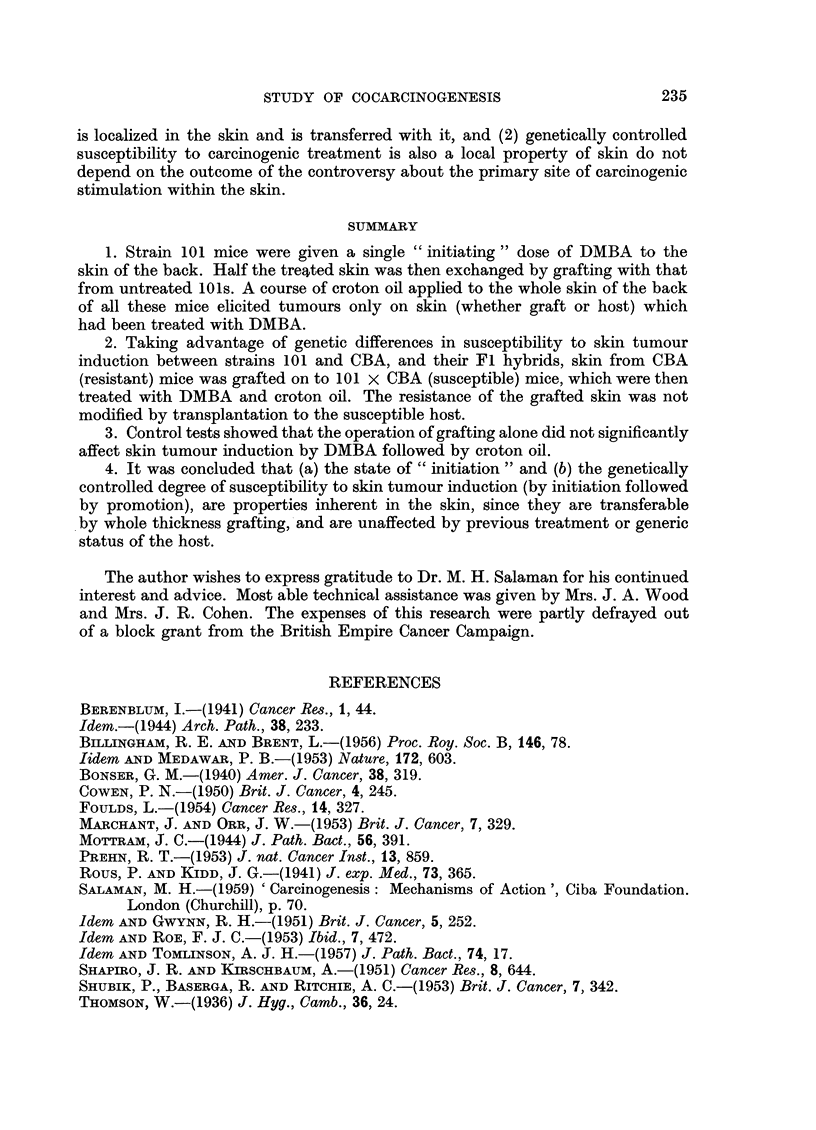# Skin Grafting in the Study of Cocarcinogenesis

**DOI:** 10.1038/bjc.1959.30

**Published:** 1959-06

**Authors:** P. N. Cowen

## Abstract

**Images:**


### SKIN GRAFTING IN THE STUDY OF COCARCINOGENESIS

P. N. COWEN

*From the Department of Cancer Research, The London Hospital Medical College, E.1**

Received for publication April 3, 1959

THE significance of the two-stage theory of carcinogenesis (e.g. Rous and Kidd, 1941 ; Berenblum, 1941, 1944 ; Mottram, 1944 ; Shubik, Baserga and Ritchie, 1953 ; Foulds, 1954) is widely recognised, although the range of species and tissues in which a two stage process has been demonstrated is narrow, and its mechanism is still far from being fully explained. We do not know, for instance, whether the essential changes which are manifested as " initiation " and " promotion " occur in the tissue in which tumours arise, or in some other part of the organism, nor whether the genetically determined differences in susceptibility to carcinogenesis are local or systemic characters.

An attempt has been made to gain some insight into these problems, in the case of skin carcinogenesis in mice.

Mouse skin after treatment with an initiating agent may appear both histologically and cytologically normal, e.g. at any time after one or more applications of urethane (Salaman and Roe, 1953), or may show only " occasional local epithelial thickening and a slight increase of keratin " e.g. 12 weeks after a single application of a carcinogenic hydrocarbon (Salaman and Gwynn, 1951), yet it behaves quite differently from untreated skin when subsequently treated with a promoting agent. It would be interesting to know whether this altered behaviour depends on a change in the skin itself, or in the rest of the animal, or in both. Orr and his collegues (Marchant and Orr, 1953) have brought forward evidence to show that the dermis plays an essential part in skin carcinogenesis, and hold the view that changes in the epidermis, the tissue from which the tumours eventually arise, are secondary. In the present work no attempt has been made to distinguish between the parts played by dermis and epidermis, but attention has been directed to the relation between the skin as a whole and the rest of the organism.

It is well-known that there are marked differences between different strains of mice in their susceptibility to carcinogenesis in various tissues (Bonser, 1940 ; Cowen, 1950 ; Salaman, 1959). In some cases, e.g. that of urethane-induced lung adenomas in the mouse (Schapiro and Kirschbaum, 1951) it seems clear that susceptibility resides in the tissue in which tumours arise and that the influence of the host was unimportant. The present work was undertaken to investigate skin carcinogenesis from a similar point of view ; to discover whether the susceptibility of the skin is a property of that tissue, or dependent on influences exerted by the rest of the animal.

The technique of whole-thickness skin-grafting was used in these studies.

* Present address : Department of Pharmacology, Guy's Hospital Medical School, London, S.E.1.

### MATERIALS AND METHODS

*Mice*.—The mice used were of 101 and CBA strains, bred in this laboratory by brother-sister mating. The former are much more susceptible than the latter to the induction of skin tumours by the standard tumour-initiating and promoting techniques used in this laboratory (Salaman, 1959). $F_1$ hybrids from both reciprocal crosses between these strains were found to have a susceptibility to the development of skin tumours intermediate between that of the parent strains. In the present work 101 ♂ × CBA ♀ $F_1$ hybrids of both sexes were used. All experiments were done on approximately equal numbers of mice of both sexes with the exception of Control Test 1(c) where all the mice were CBA males. The mice were fed on a caked diet prepared according to the Rowett Institute formula (Thomson, 1936), with water *ad libitum*. All mice were vaccinated with sheep lymph when 6–8 weeks old as a precaution against ectromelia (Salaman and Tomlinson, 1957), and only positive reactors were used. At the beginning of the experiments the mice were 8–10 weeks old.

*Chemicals and their application*.—Initiating treatment: 0·2 ml. of a 0·15 per cent (w/v) solution of 9 : 10-dimethyl-1 : 2-benzanthracene. (DMBA) in acetone (AR) was applied from a graduated 1 ml. pipette to the skin of the back after the hair had been clipped. Further treatment was delayed for 3 weeks, so that no appreciable DMBA should contaminate the recipients of grafts.

Promoting treatment: 0·3 ml. of a 0·1 per cent (v/v) solution of croton oil in acetone was applied weekly for 18 weeks.

Papillomas were counted weekly (occasionally missing a week) and in most cases the count at the 19th week was taken as a measure of tumour incidence for purposes of comparison. Croton oil was obtained from Messrs. Stafford Allen & Sons Ltd., 20 Wharf Road, N.1.

DMBA was obtained from Messrs. Light & Co. Ltd.

*Grafting technique*.—The method was based on that of Prehn (1953). The mice to be grafted had the hair clipped from their backs. They were anaesthetised with ether, and secured with surgical strapping to a warm operating table. A strictly sterile technique was not observed, but all the instruments were boiled, and sutures were kept in spirit and rinsed in sterile Ringer before use. A midline incision was made in the skin of the back, extending from the nape of the neck to the root of the tail, its ends extended transversely to one side, and these extensions joined by a fourth incision along the lateral line of the flank. About half of the skin of the back was thus removed. In the later experiments a thin plastic sheet was used as a pattern, so that pieces of skin to be cross-grafted were as nearly as possible the same size and shape. The dimensions of this pattern are shown in Fig. 1. Bleeding points soon dried spontaneously. Raw areas were temporarily covered with sterile gauze soaked in warm sterile Ringer's solution. The graft was transferred to a petri dish containing a few sheets of filter paper moistered with Ringer's solution, epithelial surface down. The panniculus carnosus was partly peeled, partly scraped off in one piece with a blunt knife. (This procedure was personally suggested by Dr. Billingham, and was found to increase the " take " rate).

The grafts were sutured in position with cotton, using one suture at each corner, one or two at upper and lower margins, three or four in the mid-line, and two at the lateral margin. To use more than this number of stitches probably imperilled the grafts, presumably because of interference with vascularisation from the margin.

The mice were grafted in pairs of the same sex. Pieces of skin were taken from one side of one mouse, rotated so that the direction of hair growth was reversed, and placed on a denuded area on the other side of the other mouse, and vice versa. This was done for two reasons ; to help show the limits of the graft (in practice the fine linear scar gave a good indication of this) and to show with certainty, by the direction of hair growth, that the graft had " taken ". A successful graft is shown in Fig. 2., 4 weeks after the operation, before re-clipping the hair for treatment.

FIG. 1.—Either the right or left half of the pattern represents the area of skin removed from the back of the mouse.

No further dressing or treatment of any kind was found necessary. The mice were placed together (up to 8 in a box) after the operation, and by the second or third week almost all stitches had fallen out or had been nibbled away.

The mice were always left for four weeks, to allow thorough healing, before any substance was applied to the skin.

### RESULTS

Although it has previously been shown that 101 and CBA mice are, respectively, susceptible and resistant to the development of skin tumours by initiation and promotion (Salaman, 1959), it was decided for the purpose of the present experiment to repeat the test on CBA mice and to show that grafting alone did not influence the response of these mice and their hybrids to initiation and promotion.

*Control tests* (a).—Thirteen male and thirteen female CBA mice were given the initiating application of DMBA and, after 3 weeks, 18 weekly applications of croton oil. The results are shown in Table I (*a*) together with comparable figures for the other strains as published elsewhere (Salaman, 1959).

(b). Twelve male and twelve female CBA mice were cross-grafted, and after waiting for four weeks for the grafts to become well established, they were given the same initiating and promoting treatment. The results of these tests are shown in Table I (*b*). It is clear that CBAs are very much more resistant to this form of carcinogenesis than 101s, and that the operation of grafting does not materially alter their response. The number of tumours as shown were the maximum present at any time during the experiment.

(c). Twenty-four male 101 mice were cross-grafted. One month later they were given the initiating application of DMBA, and 3 weeks after that promotion with croton oil was started. The first tumours appeared after 4 weekly applications of croton oil, and increased in number until the end of the experiment, when all mice had tumours. Two mice did not survive the experiment and in another two the grafts did not take, these four mice are excluded. The results are recorded in Table I (*c*).

(d). Twelve male and 12 female 101 mice were given the initiating application of DMBA. Three weeks later all mice were cross-grafted in pairs. One male and 2 females died under the anaesthetic. All the grafts took. Four weeks after grafting, promoting treatment with croton oil was begun. Tumours started to appear on both the graft and the host skin after 3 weekly applications of croton oil, and increased in number thereafter till the end of the experiment. After 18 weeks of croton oil treatment, all mice had tumours. The results are recorded in Table I (*d*).

TABLE I.—*Results of Control Tests. Skin Tumour Incidence of Mice of Various Strains and the Effect on it of Isologous Skin Grafting. There were approximately Equal Numbers of Both Sexes in Each Group Except* (c), *Where all the Mice were Males.*

| Test | Strain of mice | Number of mice | Number of tumours after 18 weeks of croton oil with the exception as shown: On host skin | On graft | On suture lines |
|---|---|---|---|---|---|
| (*a*) | CBA ungrafted | 26 | 5 | — | — |
| * | CBA ,, | 20 | 0 | After 12 | |
| * | 101 ,, | 35 | 390 | weeks of | |
| * | 101 × CBA ungrafted | 65 | 139 | croton oil | |
| (*b*) | CBA cross-grafted before initiation | 20 | 3 | 0 | 0 |
| (*c*) | 101 ,, ,, ,, | 20 | 206 | 64 | 19 |
| (*d*) | 101 cross-grafted after initiation | 21 | 278 | 83 | 14 |

* From experiments described elsewhere (Salaman, 1959).

All the tumours referred to in this paper were papillomas, and were similar to those reported in previous studies in their appearance, and tendency to regress—particularly after croton oil was stopped. A number of sections made post mortem showed the usual histological appearances.

In this and later experiments, tumours which arose on the suture line and which could therefore neither be ascribed with certainty to the skin of the graft nor to that of the host mouse, were counted and recorded separately.

In tests (c) and (d), tumour incidence on the grafted part of the skin was appreciably less than on the host skin. The area of the graft was, in fact, slightly less than half the treated area and it is unlikely that the operation of grafting either before or after the application of the initiator materially altered tumour incidence.

### *Experiment I*

*Grafting of initiated skin on to non-initiated mice and* vice versa

Thirty-six 101 mice (eighteen males and eighteen females) were given the initiating application of DMBA. After three weeks they were cross-grafted with 36 untreated mice of the same strain and sex. Thus there were 36 DMBA-treated mice with untreated skin grafts and 36 untreated mice with DMBA-treated grafts. Four weeks after grafting all mice received croton oil weekly for 18 weeks.

Tumour incidence is recorded in Table II. Fig. 3 shows a DMBA-treated mouse grafted with untreated skin, after subsequent treatment with croton oil. The graft and tumours on the host skin and on the suture line, are visible.

TABLE II.—*Tumour Development in Skin Grafts and Host Skin after Cross-grafting Between " Initiated " and "Non-initiated" Isologous Mice, with Subsequent " Promotion ".*

| Number of cross-grafted 101 mice | Number of survivors with successful grafts | Number of tumours on: Host skin | Graft | Suture line |
|---|---|---|---|---|
| 36 " initiated " mice | 25 | 170 | 2 | 11 |
| 36 " non-initiated " mice | 25 | 1 | 33 | 9 |

There were equal numbers of males and females in each group; cross-grafted pairs were of the same sex.

### *Experiment* 2

*Grafting of resistant skin on to susceptible mice*

Twelve male and 12 female 101 × CBA hybrids had the skin on one half of their backs replaced by skin taken from CBAs. The CBA mice were sacrificed for this purpose and enough skin was obtained to graft the halves of two hybrids, whose skin was discarded. Otherwise the method was essentially the same as already described. All grafts took. After four weeks, the usual course of initiation and promotion was started. The results are shown in Table III (*a*) and represent the maximum numbers of tumours seen at any time during the experiment.

The experiment was repeated on 17 male and 15 female hybrids using much larger pieces of skin from the donor (CBA) mice, which covered nearly the whole of the backs. All grafts took. Further treatment was as before. Apart from one occasion when 2 extra transient tumours were seen on suture lines these numbers

EXPLANATION OF PLATES.

FIG. 2.—A mouse four weeks after grafting by the method described.
Note reversed direction of hair growth on the graft.

FIG. 3.—DMBA-treated mouse grafted with untreated skin and then given croton oil. The graft is on the right—the caudal parts of the suture line and the reversed direction of hair growth are noticeable. Two tumours in the middle of the back are on the host skin, the third is on the suture line. Another tumour arising from the suture line is seen near the extreme caudal point of the graft.

2

3

represent the maximum number seen during the experiment. The results are shown in Table III (*b*).

TABLE III.—*Tumour Development in Skin from CBA (Resistant) Mice when Grafted to* 101 × *CBA Hybrids (Susceptible) after Subsequent "Initiation" and "Promotion".*

| | Number of survivors with successful grafts | Number of tumours on: Host skin | Graft | Suture line |
|---|---|---|---|---|
| (*a*) 24 hybrids with half of back grafted | 23 | 47 | 0 | 7 |
| (*b*) 32 hybrids with whole of back grafted | 32 | 7 | 0 | 1 |

Both sexes were approximately equally represented in each group.

It was apparent that grafts of skin of resistant mice which were transferred to susceptible mice remained resistant to the development of tumours.

No stitch abscesses, or other evidence of sepsis was ever seen at any time as a result of the operation of skin grafting.

No apparent sex difference in tumour response was noted in any of the experiments reported, and the results for both sexes have therefore been pooled.

### DISCUSSION

It was early realized that differences between tumour incidence in different groups of treated mice, or between grafted and host skin, must be large if any conclusions were to be drawn from them. Previous experience made it improbable that small differences in average tumour counts would be statistically significant, because of individual variation. There were also unavoidable uncertainties connected with the method, e.g. possible stretching or shrinkage of grafts, and the difficulty of evaluating tumours which appeared on heads and limbs of host mice ; areas not represented on the grafts. Fortunately the results were sufficiently clear-cut to allow certain definite deductions to be made.

The control series (Table I), shows the following :

(1) Susceptibility to skin tumour induction by initiation and promotion is much greater in 101 than in CBA mice.

(2) Susceptibility in 101 × CBA hybrids is intermediate between that of the parent strains.

(3) Isologous grafting of half the dorsal skin, either before initiation (in 101 and CBAs) or between initiation and promotion (in CBAs) does not appreciably alter tumour incidence (taking into consideration the fact that the final area of the graft was always slightly less than half the treated area of the back).

The results of isologous cross-grafting between " initiated " and " non-initiated " 101s (Experiment 1 and Table II) show that " initiation " is inherent in the skin, at any rate at 3 weeks after the application of the initiator. The normal host did not alter the tumour incidence on the " initiated " graft, nor did the normal graft alter that on the " initiated " host, when both were subsequently treated with the promoting agent. With the exception of a very few tumours which are liable to result from the action of croton oil alone, tumours arose only on initiated skin.

Exactly how the skin becomes initiated is not revealed by this work. Whether the DMBA acts directly on the epithelial cells or through a complex metabolic pathway is still unknown. Nevertheless we may say that by three weeks after initiation, the changes which produce the " latent cell " are complete, and the skin then behaves autonomously as far as its tumour producing propensities are concerned. It would be difficult to determine by grafting how soon after initiation with DMBA these changes occur, since the presence of excess DMBA on the treated skin might contaminate adjacent non-initiated skin if grafting were attempted too early. We can rule out contamination by the initiator in the present experiments since no tumours appeared on non-initiated skin.

The results of grafting resistant CBA skin on to susceptible 101 × CBA hybrids were also definite (Experiment 2 and Table III). Tumour incidence on the hybrid hosts was unaffected by the presence of the CBA grafts, and the grafts remained as insusceptible as they would have been if treated in their original position. Thus it appears that genetic differences in susceptibility to skin carcinogenesis by initiation and promotion depend on inherent properties of the skin itself.

For completeness, it would be desirable to graft susceptible skin on to a resistant host. Although hybrid mice are relatively more susceptible than their resistant parent, they are appreciably more resistant than their susceptible parent (Salaman, 1959). 101 skin could be grafted on to 101 × CBA hybrids, but it is unlikely that significant differences in incidence between grafted and host skin would be observed. It might also be possible to graft susceptible skin to a resistant mouse of a different strain after producing actively acquired tolerance of the foreign tissue (Billingham, Brent and Medawar, 1953; Billingham and Brent, 1956) by injecting cells of the donor strain into the future host mice *in utero* or when newborn. The method would be laborious because a low survival rate, and possibly a low success rate of grafting, would make it necessary to use very large numbers of animals.

A scrutiny of the results of the control experiments showed that the variation in tumour response between mice of the same strain (101) is due to environmental factors and not to inherent differences in the mice. The tumour yield of individual mice was studied and no correlation was found between the incidence of tumours on the mouse's own skin and the incidence on that part of it which was grafted on to another mouse. Neither was there a correlation between the incidence of tumours on the mouse's own skin and the incidence on the adjacent graft from another mouse. This is in keeping with the conclusion derived from genetic studies that the heterogeneity of response of individual mice of an inbred strain to carcinogenic treatment is due to environmental factors. Had the tumour response of each mouse depended on its genetic make-up, the correlations sought would have been found. The converse does not apply, for had these correlations been found the operation of environmental factors before grafting could not have been excluded.

Marchant and Orr (1953), on the basis of experiments in which isolated epidermal sheets were grafted, concluded that the action of carcinogens is primarily on the dermis, and that epidermal changes are secondary. The skin which was grafted in the experiments described here was of full thickness and certainly included the whole of the fibrous layer and part of the areolar layer of the dermis. Consequently the results are not relevant to the question considered by Orr and is colleagues. The conclusions reached, namely that (1) the condition of initiation is localized in the skin and is transferred with it, and (2) genetically controlled susceptibility to carcinogenic treatment is also a local property of skin do not depend on the outcome of the controversy about the primary site of carcinogenic stimulation within the skin.

### SUMMARY

1. Strain 101 mice were given a single " initiating " dose of DMBA to the skin of the back. Half the treated skin was then exchanged by grafting with that from untreated 101s. A course of croton oil applied to the whole skin of the back of all these mice elicited tumours only on skin (whether graft or host) which had been treated with DMBA.

2. Taking advantage of genetic differences in susceptibility to skin tumour induction between strains 101 and CBA, and their F1 hybrids, skin from CBA (resistant) mice was grafted on to 101 × CBA (susceptible) mice, which were then treated with DMBA and croton oil. The resistance of the grafted skin was not modified by transplantation to the susceptible host.

3. Control tests showed that the operation of grafting alone did not significantly affect skin tumour induction by DMBA followed by croton oil.

4. It was concluded that (a) the state of " initiation " and (*b*) the genetically controlled degree of susceptibility to skin tumour induction (by initiation followed by promotion), are properties inherent in the skin, since they are transferable by whole thickness grafting, and are unaffected by previous treatment or generic status of the host.

The author wishes to express gratitude to Dr. M. H. Salaman for his continued interest and advice. Most able technical assistance was given by Mrs. J. A. Wood and Mrs. J. R. Cohen. The expenses of this research were partly defrayed out of a block grant from the British Empire Cancer Campaign.

### REFERENCES

BERENBLUM, I.—(1941) *Cancer Res.*, **1**, 44.
*Idem.*—(1944) *Arch. Path.*, **38**, 233.
BILLINGHAM, R. E. AND BRENT, L.—(1956) *Proc. Roy. Soc.* B, **146**, 78.
*Iidem* AND MEDAWAR, P. B.—(1953) *Nature*, **172**, 603.
BONSER, G. M.—(1940) *Amer. J. Cancer*, **38**, 319.
COWEN, P. N.—(1950) *Brit. J. Cancer*, **4**, 245.
FOULDS, L.—(1954) *Cancer Res.*, **14**, 327.
MARCHANT, J. AND ORR, J. W.—(1953) *Brit. J. Cancer*, **7**, 329.
MOTTRAM, J. C.—(1944) *J. Path. Bact.*, **56**, 391.
PREHN, R. T.—(1953) *J. nat. Cancer Inst.*, **13**, 859.
ROUS, P. AND KIDD, J. G.—(1941) *J. exp. Med.*, **73**, 365.
SALAMAN, M. H.—(1959) ' Carcinogenesis : Mechanisms of Action ', Ciba Foundation. London (Churchill), p. 70.
*Idem* AND GWYNN, R. H.—(1951) *Brit. J. Cancer*, **5**, 252.
*Idem* AND ROE, F. J. C.—(1953) *Ibid.*, **7**, 472.
*Idem* AND TOMLINSON, A. J. H.—(1957) *J. Path. Bact.*, **74**, 17.
SHAPIRO, J. R. AND KIRSCHBAUM, A.—(1951) *Cancer Res.*, **8**, 644.
SHUBIK, P., BASERGA, R. AND RITCHIE, A. C.—(1953) *Brit. J. Cancer*, **7**, 342.
THOMSON, W.—(1936) *J. Hyg., Camb.*, **36**, 24.